# Effect of new polyherbal formulations DF1911, DF2112 and DF2813 on CFA induced inflammation in rat model

**DOI:** 10.1186/s12906-017-1711-6

**Published:** 2017-04-04

**Authors:** Bhagyashri Nagarkar, Suresh Jagtap

**Affiliations:** Department of Herbal Medicine, Interactive Research School for Health Affairs (IRSHA), Bharati Vidyapeeth Deemed University, Pune-Satara Road, Pune, Maharashtra 411043 India

**Keywords:** Anti-inflammatory, New poly-herbal formulations, *Dashamoola Kwatha*, Complete Freund’s Adjuvant

## Abstract

**Background:**

Aim of the present study was to evaluate anti-inflammatory activity of newly developed polyherbal formulations DF1911, DF2112 and DF2813. These newly developed formulations are modifications of *Dashamoola,* a well known Ayurvedic formulation, along with addition of new plants.

**Methods:**

Complete Freund’s adjuvant (CFA) induced inflammation in rat was used as an experimental model. Effects of the treatment in rats were monitored by physiological and biochemical parameters, histopathology and through gene expression studies.

**Results:**

Diclofenac sodium showed maximum percentage inhibition (56.8 ± 3.5%) of paw edema followed by *Dashamoola Kwatha* (19.9 ± 1.8%). Among test formulations treated groups, DF1911 at 250 mg/kg bw (48.2 ± 5.4%, *p* < 0.001) and DF2112 at 250 mg/kg bw (49.9 ± 3.5%, *p* < 0.001) showed significant and maximum increase in percentage inhibition of paw edema as compared to *Dashamoola Kwatha*. Hematological alterations in CFA rats were normalized after treatment with test formulations. Results of serum markers and histopathological observations also supported the activity of formulations. Increased MDA levels in liver tissue of CFA injected animals significantly (*p* < 0.05) decreased by Diclofenac sodium and test formulation treated groups. DF1911, DF2112 and DF2813 showed down-regulation of IL1-β (~6.4-fold, ~5.2-fold and ~7.6-fold), IL-6 (~1.1-fold, ~1.6-fold and ~1.9-fold), TNF-α (~2.0-fold, ~4.6-fold and ~3.5-fold), and iNOS (~1.2-fold, ~1.8-fold and ~1.1-fold) in inflamed paw tissue compared to negative control group, respectively.

**Conclusions:**

The anti-inflammatory effects of DF1911 and DF2112 in rats were significantly higher than the *Dashamoola Kwatha* and are comparable to Diclofenac sodium.

## Background

Inflammation is a local response to cellular injury marked by capillary dilatation, leukocyte infiltration, redness, heat, pain, swelling, and often loss of function and serves as a mechanism initiating the elimination of noxious agents and damaged tissue [1]. It is body’s natural defence mechanism which plays a central role in disease and aging. Though complex biological response of inflammation is a protective attempt towards harmful stimuli such as pathogens, damaged cells or irritants, it also initiates healing process for target tissue [2, 3]. There is an association between inflammation and oxidative stress. Triggering inflammatory response through damage to membrane lipids by reactive oxygen species, such as peroxide, superoxide anion, hydroxyl radical and singlet oxygen indicates its close physiological relationship with oxidative stress [4].

Management of inflammation is critical for health. The local inflammatory responses initiated by immune system are crucial for body homeostasis. Inflammation is an integral part of most of the pathological conditions that involve neutrophils, platelets, macrophages, endothelial cells and the coagulation and complement systems. Although inflammation serves a protective and healing role, chronic inflammation frequently becomes health problems. Available therapies, like Steroids and Non-Steroidal Anti-inflammatory Drugs (NSAIDs), have several adverse side effects like damage to the gastrointestinal tract, heartburn, nausea, gastric and duodenal ulcers etc [5, 6]. Therefore, the search for new anti-inflammatory drugs and identification of effective therapies is still an important field in drug discovery.

In Ayurveda, *Dashamoola*, a combination of ten plants is routine practiced for management of different inflammatory ailments [7, 8]. In our earlier studies, we have shown comparative in vivo efficacy of *Dashamoola* plants against inflammation [9] and comparative anti-inflammatory potential of different dosage forms of *Dashamoola* [10]. On the basis of these studies, different mathematical combinations of ingredient plants of *Dashamoola* along with routinely used anti-inflammatory plants viz. *Curcuma longa* (Haldi), *Pongamia pinnata* (Karanj), *Asparagus racemosus* (Shatavari) and *Terminalia chebula* (Hirda) were prepared. Three combinations viz. DF1911, DF2112 and DF2813 showing highest in vitro free radical quenching and anti-inflammatory activity were selected for further studies.

In the present study we investigated the effects of polyherbal formulations viz. DF1911, DF2112 and DF2813, developed at our institute for their anti-inflammatory action. The herbs used in the proposed formulations are known to possess anti-inflammatory, anti-allergic and wound healing properties [9, 11, 12]. Injection of CFA into the footpad of rats mimics more closely to persistent injury [13]. Therefore aspect of the study is to understand anti-inflammatory action of selected herbal formulations through modulation of inflammatory gene expression in CFA induced inflammation in rats as a model.

## Methods

### Reagents

Chemicals and suppliers: Complete Freund’s Adjuvant (Sigma, USA), Carboxy methyl cellulose (Merck, India), Diclofenac sodium (Reactin-100 SR, Cipla, India). All other chemicals used in this study were of Analytical grade.

### Plant material

Plant material was collected from natural habitats except for *Curcuma longa* L. which was collected from local markets. The plants were taxonomically identified, authenticated and voucher specimens were deposited in the herbarium of Medicinal Plants Conservation Centre, Pune (MPCC) (Table 1). Plant material was shade dried, cut into small pieces and pulverized using a mechanical grinder. The powder was passed through 80-mesh sieve and stored in an air-tight container for further use. *Curcuma longa* L. was authenticated at college of Ayurveda, Bharati Vidyapeeth University, Pune.

**Table 1:** Plants used in the formulations

Botanical name	Sanskrit name	Family	Herbarium number	Plant part used
*Aegle marmelos* (L.) Corr.	Bilva	Rutaceae	MPCC3591	Root
*Asparagus racemosus* Willd.	Shatavari	Asparagaceae	MPCC 1558	Root
*Curcuma longa* L.	Haridra	Zingiberaceae	--	Rhizomes
*Desmodium gangeticum* (L.) DC.	Shalparni	Fabaceae	MPCC 146	Root
*Gmelina arborea* Roxb.	Gambhari	Verbenaceae	MPCC 783	Root
*Oroxylum indicum* Vent.	Shyonak	Bignoniaceae	MPCC 3592	Root
*Pongamia pinnata* (L.) Pierre	Karanjah	Fabaceae	MPCC 3593	Stem bark
*Premna obtusifolia* R. Br.	Agnimantha	Verbenaceae	MPCC 2593	Root
*Solanum anguivi* Lam.	Bruhati	Solanaceae	MPCC 2627	Root
*Solanum virginianum* L.	Laghukantakari	Solanaceae	MPCC 2762	Root
*Stereospermum colais* Mabb.	Patala	Bignoniaceae	MPCC 90	Root
*Terminalia chebula* Retz.	Haritaki	Combretaceae	MPCC 3507	Fruit
*Tribulus terrestris* L.	Gokshur	Zygophyllaceae	MPCC 1097	Root
*Uraria picta* (Jacq.) Desv. ex DC.	Prishniparni	Fabaceae	MPCC 2645	Root

### Formulation preparation

Plant material was taken in the proportions as mentioned in Table 2. Formulations in the form of ‘Kwatha’ were prepared as per the standard procedure mentioned in Ayurvedic Formulary of India [14], by taking 1 part of the mixture of plants in 16 parts of water. The mixture was boiled till 1/8th of its volume and filtered through muslin cloth.

**Table 2:** Formulation composition (ratio)

	AM	AR	CL	DG	GA	OI	PP	PO	SA	SV	SC	TC	TT	UP
DF1911	1	0	0	2	1	2	0	2	1	1	1	13.02	1	1
DF2112	2	6.01	0	1	1	1	0	2	1	1	1	6.01	1	1
DF2813	2	0	3.25	1	1	2	2.45	1	1	1	1	7.33	1	2

*DF Dashamoola* formulation, *AM A. marmelos, AR A. racemosus, CL C. longa, DG D. gangeticum, GA G. arborea, OI O. indicum, PO P. obtusifolia, PP P. pinnata, SA S. anguivi, SC S. colais, SV S. virginianum, TC T. chebula, TT T. terrestris, UP U. picta*

### Acute oral toxicity study

The acute toxicity study of selected test formulations was carried out according to the Organization for Economic Cooperation and Development (OECD) guideline 420 [15]. All test formulations viz. DF1911, DF2112 and DF2813 were found to be safe up to 2000 mg/kg bw orally.

### CFA induced inflammation in rats

#### Animals

The experiment was performed in Female Wistar albino rats (weighing 180-200 g) obtained from National Institute of Biosciences, Pune, India. They were kept under standard husbandry conditions and provided with food and water ad libitum. All animals were housed in polypropylene cages (43 X 27 X 15 cm) with not more than four animals per cage under standard laboratory conditions viz. 25 ± 2 °C temperature and 12 h light and dark cycle. Institutional animal ethics committee approval for the experimental protocol was obtained before initiation of the study (Ref No. BVDUMC/187/2014-2015). All the procedures were followed as per the CPCSEA norms.

### Preparation of CFA (0.5 mg/ml mycobacterium emulsion)

The bottle of CFA (1 mg/ml *Mycobacterium tuberculosis*, heat killed and dried) was vortexed prior to use to prevent sedimentation of Mycobacteria during storage. 0.5 ml CFA was added to 0.5 ml sterile 0.9% saline to obtain final 0.5 mg/ml mycobacterium emulsion [16].

### Experimental design

Total 78 animals were coded and randomly divided into 13 groups containing six animals per group. First group was Healthy control. The second group served as Negative control, which did not receive any treatment. The third group served as Positive control, which received Diclofenac sodium at a dose of 15 mg/kg bw. The fourth group was treated with routinely practised *Dashamoola* formulation (*Dashamoola Kwatha*) at a dose of 1.8 ml/kg bw. Rest of the groups were treated with the test formulations as shown in Table 3.

**Table 3:** Test groups of CFA induced inflammation in rat model

S.N.	Test groups	Treatment specification
1.	HC	-
2.	NC	CFA only (0.1 ml/paw)
3.	Diclo	CFA + Diclofenac sodium (15 mg/kg bw)
4.	Dasha	CFA + *Dashamoola Kwatha* (1.8 ml/kg bw)
5.	DF1911-LD	CFA + DF1911 (250 mg/kg bw)
6.	DF1911-MD	CFA + DF1911 (500 mg/kg bw)
7.	DF1911-HD	CFA + DF1911 (1000 mg/kg bw)
8.	DF2112-LD	CFA + DF2112 (250 mg/kg bw)
9.	DF2112-MD	CFA + DF2112 (500 mg/kg bw)
10.	DF2112-HD	CFA + DF2112 (1000 mg/kg bw)
11.	DF2813-LD	CFA + DF2813 (250 mg/kg bw)
12.	DF2813-MD	CFA + DF2813 (500 mg/kg bw)
13.	DF2813-HD	CFA + DF2813 (1000 mg/kg bw)

*HC* healthy control, *NC* negative control, *Diclo* diclofenac sodium, *Dasha Dashamoola Kwatha*, *DF Dashamoola* formulation, *LD* lower dose, *MD* middle dose, *HD* higher dose

Inflammation was induced in all groups except healthy control, by sub-planter injection of 0.1 ml of CFA (0.5 mg/ml) on right hind paw on day 1st of the experiment. Treatment with test drugs and standard drugs was started from 14th day of experiment up to 28th day. All test drugs and standard drugs were administered orally. On 29th day, all animals were sacrificed and blood as well as hind paw edematous tissue was collected for further investigations [17].

### Assessment of anti-inflammatory potential

The progression of CFA induced inflammation was evaluated by measuring the change in paw volume on day 7, 14, 21 and 28 after induction of inflammation. At the end of the experiment, change in paw volume with percentage inhibition was determined.

Percent inhibition of edema was calculated as per the following formula:

$$ \mathrm{Percent}\ \mathrm{inhibition}\ \mathrm{of}\ \mathrm{edema}=\frac{{\mathrm{V}}_{\mathrm{c}}-{\mathrm{V}}_{\mathrm{t}}}{{\mathrm{V}}_{\mathrm{c}}}\times 100 $$

Where, V_c_ = Paw volume of negative control group animal, V_t_ = Paw volume of treatment group animal.

Changes in body weight were recorded. Blood was collected in EDTA coated tubes for hematological estimations. Animals were euthanized on 29th day by cervical decapitation. Liver was excised and snap-frozen and stored at −80 °C for subsequent malondialdehyde (MDA) level determination. The marker enzymes alkaline phosphatase (ALP), serum albumin, serum glutamic-pyruvic transaminase (SGPT), serum glutamic oxaloacetic transaminase aspartate (SGOT) and serum acid phosphatase were analyzed in serum separated from blood as per the manufacturer’s instructions in commercial kits (Coral Clinical Systems, Goa, India).

### MDA levels in liver tissue

Lipid peroxidation levels in liver tissue were determined colorimetrically by measuring MDA using thiobarbituricacid-reactive substances (TBARS) [18]. Briefly, rat liver was homogenised in icecold 0.15 M KCl (10% *w*/*v*). 0.5 ml of tissue homogenate was mixed with 1 ml 0.15 M KCl and incubated for 30 min at 37 °C. The reaction was terminated by addition of 2 ml ice-cold 0.25 N HCl containing 0.19% TBA and 7.5% TCA. 200 μl of 0.5% BHT prepared in Methanol was added to this mixture. The reaction mixture was incubated at 80 °C in water bath for 60 min, cooled to room temperature and centrifuged at 5000 g for 15 min. The absorbance of the supernatant was measured at 532 nm against blank, containing all reagents except tissue homogenate. The concentration of TBARS was expressed as μM/g of tissue, using 1,1,3,3-tetramethoxypropane as a standard.

### Histopathological analysis of edematous tissue

For histopathology, the right paw (edematous paw) were removed, washed with saline and stored in 10% formalin. Tissues were dehydrated, processed and embedded in paraffin wax. 4 μm sections were prepared and stained with hematoxylin and eosin (H&E) and observed under light microscope.

### Quantitative real-time reverse transcription-polymerase chain reaction analysis

Animals were killed at the end of the experiment and paw tissues of inflammation induced rats and normal rats were removed. The paw tissues were flash frozen immediately in liquid nitrogen and stored at −80 °C for PCR studies. qPCR analysis was carried out for the middle dose treated group of each test formulation. For qPCR analysis, total RNA from isolated paw tissue was extracted using TRIZOL reagent (Sigma Aldrich, USA) with PureLink RNA mini kit (Invitrogen CA, USA). The quality of the isolated RNA was determined using agarose gel electrophoresis followed by quantification by measuring absorbance at 260 nm. The first strand cDNA was synthesized from 1 μg of total RNA using the SuperScript first-strand synthesis system for quantitative real-time PCR (Invitrogen CA, USA). The qPCR analysis was performed with the help of a StepOne real time PCR system (Applied Biosystems, CA, USA) using TaqMan gene expression assays (Applied Biosystems, CA, USA). Cycling conditions were 50 °C for 2 min; 95 °C for 10 min; and 40 cycles of 95 °C for 15 s, 60 °C for 1 min. The Taqman gene expression assays that were used in this study are IL1-β (IL1b; Rn00580432_m1), IL-6 (IL6; Rn01410330_m1), TNF-α (Tnf; Rn01525859_g1) and iNOS (Nos2; Rn00561646_m1). The data, representative of paw tissue at least from three rats, were analyzed using Data Assist software version 3.0. The relative abundance of RNA was calculated to the amount of β-actin (Actb; Rn00667869_m1) using StepOne software version 2.2.2 (Applied Biosystems, CA, USA), DataAssist version 3.0 (Applied Biosystems, CA, USA) and the ΔΔ Ct method [19].

### Statistical analysis

Data from all experiments are presented as Mean ± SEM and were compared with NC group using One-Way ANOVA followed by Dunnet Multiple comparison test. The significance level was set at *p* ≤ 0.05. The statistical program used was GraphPad Prism 5.0 Version for Windows, GraphPad Software (SanDiego, CA, USA).

## Results

After injection with CFA, rats developed visible clinical signs of inflammation characterized by edema in paw. In animals treated with the test formulations at different doses, the inflammatory response was evidently reduced.

### Body weight

Table 4 shows the changes in body weight of rats. It can be observed that in NC group, there was a least increase in body weight as compared to other groups. The animals gained weight more slowly than HC group animals. In comparison, standard groups exhibited an increase in body weight. In the groups treated with test formulations, all selected formulations caused weight gain which was comparable to that of HC group animals, thereby indicating positive effect.

**Table 4:** Mean changes in body weight

Treatment	Body weight (gm)		Mean changes in body weight (gm)
	0th day	29th day	
HC	220 ± 6.56	250 ± 20.7	30.7 ± 3.93
NC	233 ± 9.36	252 ± 27.6	19.8 ± 3.6
Diclo	217 ± 7.05	244 ± 13.9	26.7 ± 2.74
Dasha	211 ± 5.81	240 ± 16	29.2 ± 2.27
DF1911-LD	223 ± 9.16	254 ± 27.6	31 ± 3.84
DF1911-MD	208 ± 3.13	234 ± 17.6	26.7 ± 4.57
DF1911-HD	223 ± 5.21	251 ± 12.8	28.3 ± 1.94
DF2112-LD	210 ± 5.62	238 ± 17.7	27.8 ± 5.63
DF2112-MD	213 ± 4.31	242 ± 8.02	28.5 ± 4.3
DF2112-HD	218 ± 5.78	249 ± 16.5	30.5 ± 2.57
DF2813-LD	220 ± 8.43	250 ± 23.8	30 ± 4.76
DF2813-MD	220 ± 11	250 ± 28.7	29.5 ± 4.9
DF2813-HD	214 ± 7.51	246 ± 17.8	32 ± 1.97

*HC* healthy control, *NC* negative control, *Diclo* diclofenac sodium, *Dasha Dashamoola Kwatha*, *DF Dashamoola* formulation, *LD* lower dose, *MD* middle dose, *HD* higher dose. Values are expressed as Mean ± SEM; *n* = 6. Data were analysed by One-Way ANOVA followed by Dunnett’s multiple comparison test

### Paw edema

CFA injected animals exhibited a marked unilateral peripheral edema in paw, before the start of treatment with standard drug and test drugs. The paw volume of NC group animals continued to increase throughout the time course of the experiment. As there was no treatment given upto day 14 after CFA injection, the paw volume of all treated groups including standards (Diclo and Dasha), was increased and less swelling was observed after day 14 as compared to NC group animals. Table 5 shows the time course of edema after the injection of CFA and test formulations.

**Table 5:** Effect of test formulations on CFA induced rat paw edema

Groups	Paw edema (ml)			
	Day 7	Day 14	Day 21	Day 28
HC	0.06 ± 0.02***^&&&$$$^	0.05 ± 0.01***^&&&$$$^	0.03 ± 0.01***^$$$^	0.04 ± 0.02***^$$$^
NC	0.98 ± 0.1^###^	0.79 ± 0.07^###^	0.81 ± 0.07^###&&^	0.8 ± 0.06^###&&^
Diclo	0.88 ± 0.06^###^	0.66 ± 0.04^###^	0.32 ± 0.03**^$$^	0.35 ± 0.05**
Dasha	0.95 ± 0.08^###^	0.78 ± 0.12^###^	0.81 ± 0.06^###&&^	0.64 ± 0.03^###^
DF1911-LD	0.98 ± 0.05^###^	0.73 ± 0.04^###^	0.52 ± 0.06^##^	0.39 ± 0.06^#^**
DF1911-MD	0.88 ± 0.04^###^	0.74 ± 0.08^###^	0.51 ± 0.05^##^	0.43 ± 0.05^#^*
DF1911-HD	0.96 ± 0.06^###^	0.78 ± 0.05^###^	0.69 ± 0.08^###&^	0.54 ± 0.11^###^
DF2112-LD	0.86 ± 0.09^###^	0.86 ± 0.12^###^	0.53 ± 0.05^##^	0.41 ± 0.05^#^**
DF2112-MD	1 ± 0.09^###^	0.79 ± 0.06^###^	0.7 ± 0.11^###^	0.52 ± 0.11^##^
DF2112-HD	0.97 ± 0.13^###^	0.84 ± 0.13^###^	0.67 ± 0.15^###^	0.53 ± 0.07^##^
DF2813-LD	0.88 ± 0.07^###^	0.82 ± 0.1^###^	0.66 ± 0.13^###^	0.62 ± 0.13^###^
DF2813-MD	0.98 ± 0.1^###^	0.78 ± 0.04^###^	0.72 ± 0.09^###&^	0.7 ± 0.08^###&^
DF2813-HD	1.1 ± 0.08^###^	0.89 ± 0.09^###^	0.82 ± 0.15^###&&^	0.87 ± 0.12^###&&&^

*HC* healthy control, *NC* negative control, *Diclo* diclofenac sodium, *Dasha Dashamoola Kwatha*, *DF Dashamoola* Formulation, *LD* lower dose, *MD* middle dose, *HD* higher dose. Values are expressed as Mean ± SEM (*n* = 6) using one way ANOVA followed by Dunnet’s multiple comparison test. ^#^ = *p* < 0.05, ^##^ = *p* < 0.01, ^###^ = *p* < 0.001 when compared to HC; * = *p* < 0.05, ** = *p* < 0.01, *** = *p* < 0.001 when compared to NC; ^&^ = *p* < 0.05, ^&&^ = *p* < 0.01, ^&&&^ = *p* < 0.001 when compared to Diclo; ^$$^ = *p* < 0.01, ^$$$^ = *p* < 0.001 when compared to Dasha

Diclo group, which was treated with the standard drug Diclofenac sodium, showed significant and maximum decrease in paw volume (0.35 ± 0.05 ml, *p* < 0.01) on day 28 of the experiment as compared to NC. Dasha group which was treated with *Dashamoola Kwatha* as another standard, also showed decrease in paw volume (0.64 ± 0.03 ml). Among test formulation treated groups, DF1911-LD (0.39 ± 0.06 ml, *p* < 0.05), DF1911-MD (0.43 ± 0.05 ml, *p* < 0.05), DF2112-LD (0.41 ± 0.05 ml, *p* < 0.01), DF2112-MD (0.52 ± 0.11 ml) groups showed significant decrease in paw volume as compared to the NC group. The maximum paw volume decrease was observed in DF1911-LD treated group on day 28 of the experiment (0.39 ± 0.06 ml, *p* < 0.05) as compared to NC group. DF2813 treated animals dis not display significant changes in paw edema as compared to NC group.

Administration of DF1911 and DF2112 significantly inhibited development of swelling induced by CFA thereby indicating anti-inflammatory activity which was maintained until the experiment was terminated on day 28. These results are comparable to Diclofenac sodium (15 mg/kg bw) treated group, which displayed significant (*p* < 0.01) decrease in paw edema on day 28 of the experiment. The test formulation treated groups exhibited higher decrease in paw edema than *Dashamoola Kwatha* treated group.

### Percentage inhibition of paw edema

**Fig. 1:**
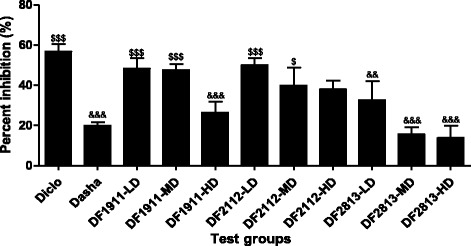
Percentage inhibition of paw edema. Diclo: Diclofenac sodium, Dasha: *Dashamoola Kwatha*, DF: *Dashamoola* Formulation, LD: Lower dose, MD: Middle dose, HD: Higher dose. Values are expressed as Mean ± SEM; *n* = 6. Data were analysed by One-Way ANOVA followed by Dunnett’s multiple comparison test. && = *p* < 0.01, &&& = *p* < 0.001 when compared to Diclo; $$$ = *p* < 0.001 when compared to Dasha

Figure 1 shows the percentage inhibition of paw edema on 28th day of the experiment. Administration of selected formulations considerably inhibited development of edema induced by CFA, thereby showing the percentage inhibition of edema comparable to that of the standard drugs treated groups.

Diclo group, which was treated with the standard drug Diclofenac sodium, showed maximum percentage inhibition of paw edema (56.8 ± 3.5%) on day 28 of the experiment. Dasha group which was treated with *Dashamoola Kwatha* as another standard also had decrease in paw volume (19.9 ± 1.8%). Among test formulation treated groups, DF1911-LD (48.2 ± 5.4%, *p* < 0.001), DF1911-MD (47.6 ± 2.9%, *p* < 0.001); DF2112-LD (49.9 ± 3.5%, *p* < 0.001), DF2112-MD (39.9 ± 9%, *p* < 0.05) displayed significant increase in percentage inhibition of paw edema when compared to Dasha group thereby indicating comparable activity to that of Diclofenac sodium. DF2813 showed insignificant activity against paw edema as compared to Dasha group.

Lower and middle doses (250 mg/kg bw and 500 mg/kg bw respectively) of test formulations used were found to be more effective than the higher dose (1000 mg/kg bw).

### Hematological alterations

Hematological alterations in CFA rats were normalized after treatment with test formulations. The CFA-induced hematological disturbance, such as decreased hemoglobin (Hb) count, decreased RBC count, increase in WBC count and increased erythrocyte sedimentation rate (ESR) were favorably altered by treatment with test formulations (Table 6).

**Table 6:** Alterations in hematological parameters in CFA-induced inflammation in rats

Groups	Haemoglobin (gm %)	RBC (10^6^/uL)	WBC total count (10^3^/cumm)	ESR (mm/h)
HC	13.3 ± 0.23^&&&^	7.82 ± 0.24**	5.07 ± 0.32	0.5 ± 0.29
NC	12.9 ± 0.18^&&^	6.19 ± 0.45^##&&&^	10.9 ± 1.77	1 ± 0.41
Diclo	10.38 ± 0.71^###^**^$^	7.9 ± 0.2***	7.54 ± 0.92	0.5 ± 0.29
Dasha	12.4 ± 0.48^&^	7.16 ± 0.19	6.27 ± 1.15	0.75 ± 0.25
DF1911-LD	13.73 ± 0.12^&&&^	8.12 ± 0.22***	6.65 ± 2.54	1.25 ± 0.25
DF1911-MD	13.95 ± 0.39^&&&^	8.09 ± 0.15***	5.01 ± 2.71	0.5 ± 0.29
DF1911-HD	12.75 ± 0.9^&&^	7.36 ± 0.54*	8.99 ± 2.6	1.25 ± 0.25
DF2112-LD	14.25 ± 0.18^&&&$^	8.27 ± 0.06***^$^	7.8 ± 2.94	0.5 ± 0.29
DF2112-MD	14.03 ± 0.39^&&&^	8.31 ± 0.24***^$^	6.1 ± 0.53	0.75 ± 0.25
DF2112-HD	14.05 ± 0.45^&&&^	8.32 ± 0.32***^$^	5.55 ± 2.85	0.5 ± 0.29
DF2813-LD	14.43 ± 0.39^&&&$^	8.27 ± 0.16***^$^	6.03 ± 1.42	0.75 ± 0.25
DF2813-MD	14.15 ± 0.18^&&&^	8.31 ± 0.07***^$^	7.4 ± 0.82	0.75 ± 0.25
DF2813-HD	14.08 ± 0.23^&&&^	8.27 ± 0.09***^$^	6.01 ± 0.23	0.75 ± 0.25

*HC* healthy control, *NC* negative control, *Diclo* diclofenac sodium, *Dasha Dashamoola Kwatha*, *DF Dashamoola* formulation, *LD* lower dose, *MD* middle dose, *HD* higher dose. Values are expressed as Mean ± SEM; *n* = 6. Data were analysed by One-Way ANOVA followed by Dunnett’s multiple comparison test. ^##^ = *p* < 0.01, ^###^ = *p* < 0.001 when compared to HC; * = *p* < 0.05, ** = *p* < 0.01, *** = *p* < 0.001 when compared to NC; ^&^ = *p* < 0.05, ^&&^ = *p* < 0.01, ^&&&^ = *p* < 0.001 when compared to Diclo; ^$^ = *p* < 0.05 when compared to Dasha

### Serum analysis

Effects of test formulations on different serum biochemical parameters are presented in Table 7. NC showed highest Total ALP activity (19.9 ± 1.27 K.A. units). Diclo group had significant (*p* < 0.01) decrease in Total ALP activity (8.98 ± 0.59 K.A. units). In case of test formulation treated groups, decrease in ALP levels was observed but the difference was insignificant as compared to NC.

**Table 7:** Effects of test formulations on biochemical parameters in CFA-induced inflammation in rats

Groups	Total ALP activity in K.A. units	Albumin in g/dl	SGOT enzyme activity (U/ml)	SGPT enzyme activity (U/ml)	Total acid phosphatase activity in K.A. units
HC	7.25 ± 1.71***^$$^	6.61 ± 0.38***^$$$^	145 ± 14.2***^$$^	53.4 ± 5.2***	5.26 ± 0.27**^$^
NC	19.9 ± 1.27^###&&^	2.66 ± 0.43^###&&&^	255 ± 34.7^###&^	123 ± 2.7^###&&&$$$^	10.8 ± 0.59^##&&^
Diclo	8.98 ± 0.59**^$^	5.38 ± 0.29***	181 ± 17.9*	54.6 ± 3.1***	5.78 ± 0.45**
Dasha	17.9 ± 1.72^##&^	3.97 ± 0.27^###^	218 ± 17.7^##^	69 ± 8.2***	9.39 ± 1.66^#^
DF1911-LD	16.4 ± 1.83^#^	4.34 ± 0.42^###^*	211 ± 8^#^	83.9 ± 7.4^#^**^&^	8.34 ± 0.66
DF1911-MD	16.4 ± 2.62^#^	3.86 ± 0.36^###&^	202 ± 13	81.2 ± 10.2**	6.11 ± 0.7**
DF1911-HD	16.6 ± 2.83^#^	3.87 ± 0.16^###&^	228 ± 21.1^##^	78.1 ± 7.2***	9.39 ± 1.66^#^
DF2112-LD	12.8 ± 1.68	4.69 ± 0.58^##^**	206 ± 18.4^#^	76.7 ± 6.2***	6.41 ± 1.48*
DF2112-MD	13.8 ± 1.35	5.4 ± 0.28***^$^	199 ± 8.5	59.1 ± 5***	6.95 ± 0.42
DF2112-HD	13.4 ± 2.57	5.38 ± 0.33***	182 ± 12.9	62.4 ± 7.6***	6.27 ± 0.38*
DF2813-LD	17.3 ± 2.12^##&^	4.36 ± 0.29^###^*	217 ± 16.1^#^	88.3 ± 7.7^##^*^&&^	8.11 ± 0.77
DF2813-MD	19.1 ± 3.2^##&^	4.72 ± 0.33**^##^	205 ± 11	93.6 ± 10.1^##&&^	8.74 ± 0.37
DF2813-HD	16.5 ± 1.51^#^	5.12 ± 0.4***^#^	211 ± 5.8^#^	96 ± 7^##&&^	9.32 ± 0.76^#^

*HC* healthy control, *NC* negative control, *Diclo* diclofenac sodium, *Dasha Dashamoola Kwatha*, *DF Dashamoola* formulation, *LD* lower dose, *MD* middle dose, *HD* higher dose, *ALP* alkaline phosphatase, *SGOT* serum glutamic-oxaloacetic transaminase, *SGPT* serum glutamic-pyruvic transaminase. Values are expressed as Mean ± SEM; *n* = 6. Data were analysed by One-Way ANOVA followed by Dunnett’s multiple comparison test. ^#^ = *p* < 0.05, ^##^ = *p* < 0.01, ^###^ = *p* < 0.001 when compared to HC; * = *p* < 0.05, ** = *p* < 0.01, *** = *p* < 0.001 when compared to NC; ^&^ = *p* < 0.05, ^&&^ = *p* < 0.01, ^&&&^ = *p* < 0.001 when compared to Diclo; ^$^ = *p* < 0.05, ^$$^ = *p* < 0.01, ^$$$^ = *p* < 0.001 when compared to Dasha

In comparison with NC, albumin levels were significantly (*p* < 0.001) increased in Diclo group (5.38 ± 0.29 g/dl). DF1911 also showed significant increase in albumin levels at 250 mg/kg dose, while DF2112 and DF2813 had significant (*p* < 0.01) increase in albumin levels at all 3 selected doses as compared to NC.

SGOT activity was significantly (*p* < 0.05) altered in Diclo group but Dasha and test formulation treated groups did not show significant changes in SGOT activity.

There was significant (*p* < 0.05) decrease in the levels of SGPT observed in DF1911 and DF2112 treated groups, however, no significant (*p* > 0.05) changes were observed in DF2813 treated groups as compared to NC.

In case of phosphatase activity, Diclo showed significant (*p* < 0.01) decrease, whereas Dasha showed no significant decrease. Out of selected test formulations, DF1911-MD (*p* < 0.01), DF2112-LD (*p* < 0.05) and DF2112-HD (*p* < 0.05) displayed a significant decrease in the phosphatase activity; while DF2813 did not display any significant decrease in the activity, as compared to NC.

### MDA levels in liver tissue

**Fig. 2:**
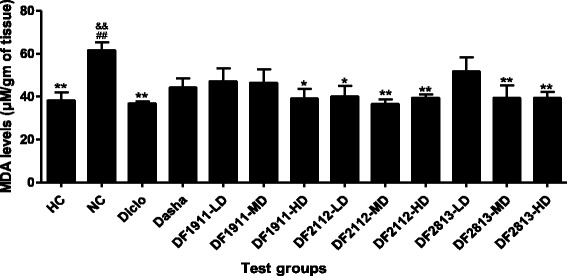
MDA levels in liver tissue. HC: Healthy control, NC: Negative control, Diclo: Diclofenac sodium, Dasha: *Dashamoola Kwatha*, DF: *Dashamoola* Formulation, LD: Lower dose, MD: Middle dose, HD: Higher dose. Values are expressed as Mean ± SEM; *n* = 6. Data were analysed by One-Way ANOVA followed by Dunnett’s multiple comparison test. * = *p* < 0.05, ** = *p* < 0.01 when compared to NC; && = *p* < 0.01 when compared to Diclo

TBARS concentrations expressed as MDA levels in the liver are shown in Fig. 2. NC group animals showed highest MDA level (61.7 ± 3.69 μM/g of tissue). There was significant decrease in MDA levels in rats treated with Diclofenac sodium, DF1911-HD (*p* < 0.05), DF2112-LD (*p* < 0.05), DF2112-MD (*p* < 0.01), DF2112-HD (*p* < 0.01), DF2813-MD (*p* < 0.01) and DF2813-HD (*p* < 0.01) as compared to NC group animals.

### Histopathological analysis

**Fig. 3:**
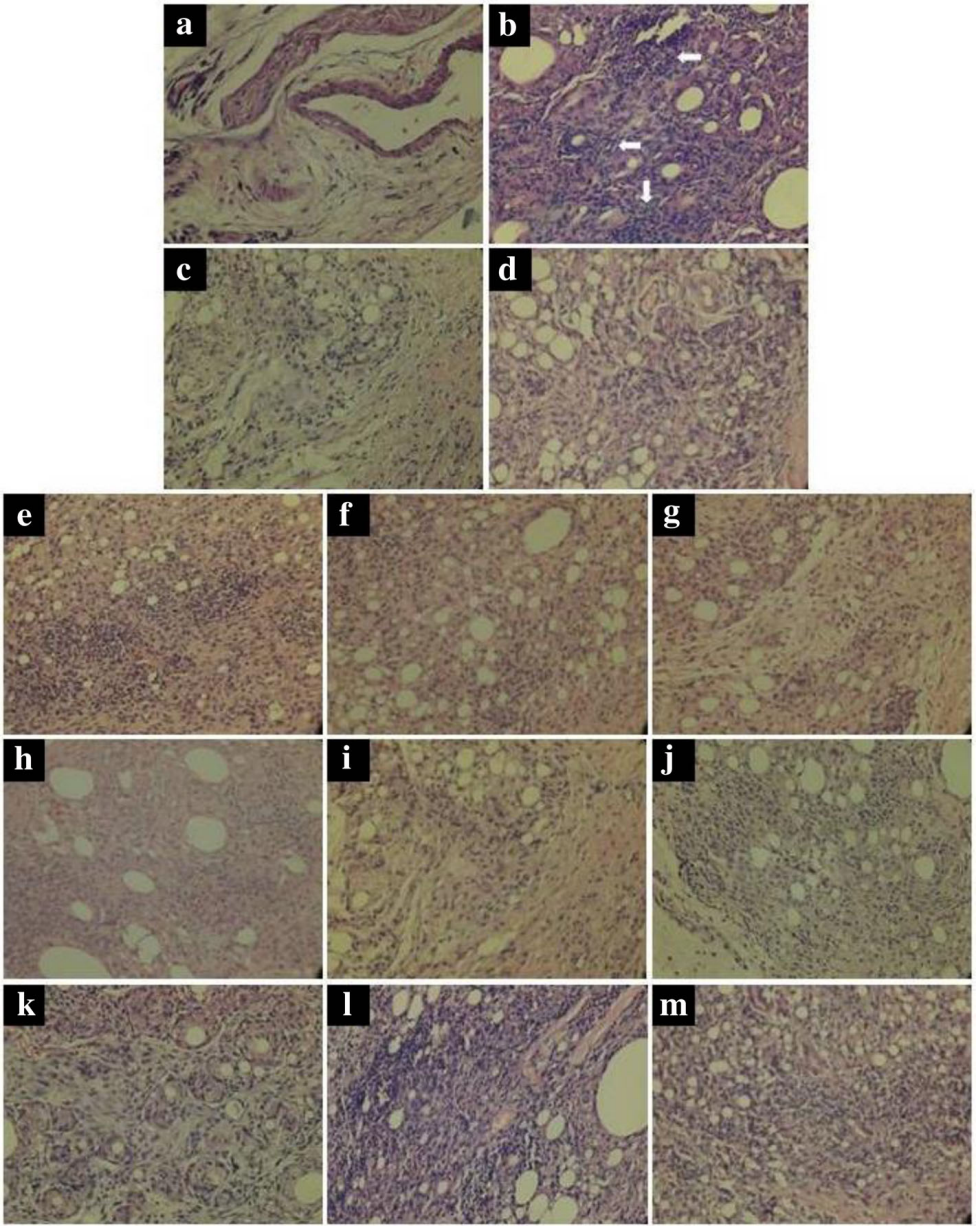
Histological sections of paw tissues. Representative histological sections of paw tissues. Samples were harvested on 29th day after CFA injection. H&E staining (40X). **a** HC, **b** NC, **c** Diclo, **d** Dasha, **e** DF1911-LD, **f** DF1911-MD, **g** DF1911-HD, **h** DF2112-LD, **i** DF2112-MD, **j** DF2112-HD, **k** DF2813-LD, **l** DF2813-MD, **m** DF2813-HD. Normal appearance of paw tissue without any lesion seen in Figure (**a**). Extensive cellular infiltrations are seen in Figure (**b**, **j**, **k**, **l** and **m**). Mild cellular infiltrations are seen in Figure (**c**, **d**, **e**, **f**, **g**, **h** and **i**). (HC: Healthy control, NC: Negative control, Diclo: Diclofenac sodium, Dasha: *Dashamoola Kwatha*, DF: *Dashamoola* Formulation, LD: Lower dose, MD: Middle dose, HD: Higher dose)

As shown in Fig. 3, there was no sign of inflammation in the paw tissue of HC group rats. Paw tissue of NC group rats revealed an inflammation with extensive cellular infiltration. Diclofenac sodium treated group exhibited mild cellular infiltration, whereas *Dashamoola Kwatha* treated group showed moderate cellular infiltration. In case of the test formulations treated groups, DF1911-LD, DF1911-MD, DF1911-HD, DF2112-LD, DF2112-MD exhibited reduced cellular infiltration as compared to NC group.

### Quantitative real-time reverse transcription-polymerase chain reaction analysis

In this analysis the expression of key cytokines viz. IL1-β, IL-6, TNF-α, and iNOS was studied as these cytokines have been reported to be expressed at significant levels during chronic inflammation [20].

**Fig. 4:**
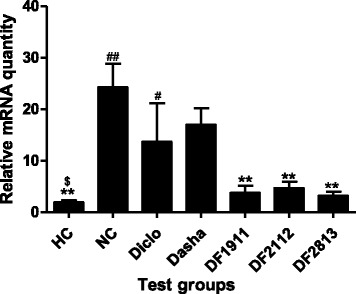
Effect of formulations on the mRNA expression of IL1-β in inflamed paw tissue. HC: Healthy control, NC: Negative control, Diclo: Diclofenac sodium, Dasha: *Dashamoola Kwatha*, DF: *Dashamoola* Formulation. Values are expressed as Mean ± SEM (*n* = 3). Comparisons were done between NC/HC and each individual treated group by Dunnett’s multiple comparison test. # = *p* < 0.05, ## = *p* < 0.01 when compared to HC; ** = *p* < 0.01 when compared to NC; $ = *p* < 0.05 when compared to Dasha

NC group rats showed significant (*p* < 0.05) up-regulation of IL1-β by ~12.8-fold, when compared to the HC. On the contrary, Diclo and Dasha showed down-regulation of IL1-β expression by ~1.8-fold and ~1.4-fold respectively. DF1911, DF2112 and DF2813 showed down-regulation of IL1-β expression by ~6.4-fold, ~5.2-fold and ~7.6-fold respectively when compared to NC (Fig. 4).

**Fig. 5:**
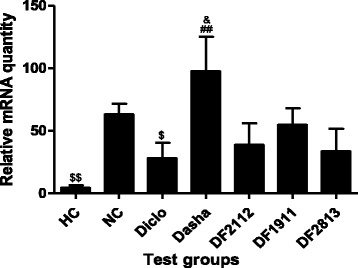
Effect of formulations on the mRNA expression of IL-6 in inflamed paw tissue. HC: Healthy control, NC: Negative control, Diclo: Diclofenac sodium, Dasha: *Dashamoola Kwatha*, DF: *Dashamoola* Formulation. Values are expressed as Mean ± SEM (*n* = 3). Comparisons were done between NC/HC and each individual treated group by Dunnett’s multiple comparison test. ## = *p* < 0.01 when compared to HC; & = *p* < 0.05 when compared to Diclo; $ = *p* < 0.05, $$ = *p* < 0.01 when compared to Dasha

In case of IL-6, NC group rats showed up-regulation of IL-6 gene expression by ~13.4-fold as compared to HC, whereas Diclo and Dasha showed down-regulation by ~2.2-fold and ~0.6-fold respectively. DF1911, DF2112 and DF2813 showed down-regulation of IL-6 gene expression by ~1.1-fold, ~1.6-fold and ~1.9-fold respectively when compared to NC (Fig. 5).

**Fig. 6:**
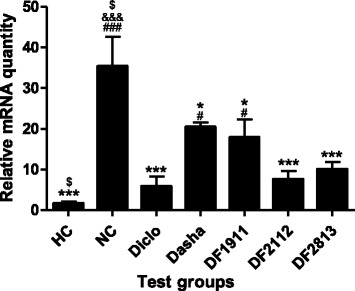
Effect of formulations on the mRNA expression of TNF-α in inflamed paw tissue. HC: Healthy control, NC: Negative control, Diclo: Diclofenac sodium, Dasha: *Dashamoola Kwatha*, DF: *Dashamoola* Formulation. Values are expressed as Mean ± SEM (*n* = 3). Comparisons were done between NC/HC and each individual treated group by Dunnett’s multiple comparison test. ### = *p* < 0.001 when compared to HC; * = *p* < 0.05, *** = *p* < 0.001 when compared to NC; &&& = *p* < 0.001 when compared to Diclo; $ = *p* < 0.05 when compared to Dasha

TNF-α gene expression was found to be up-regulated in NC group rats by ~22.1-fold as compared to HC, whereas Diclo and Dasha showed down-regulation by ~6.0-fold and ~1.7-fold respectively. DF1911, DF2112 and DF2813 showed down-regulation of TNF-α gene expression by ~2.0-fold, ~4.6-fold and ~3.5-fold respectively when compared to NC (Fig. 6).

**Fig. 7:**
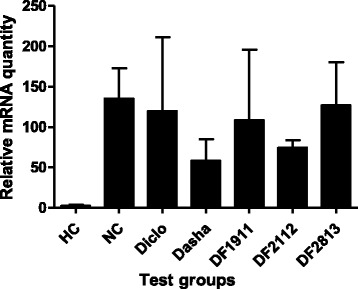
Effect formulations on the mRNA expression of iNOS in inflamed paw tissue. HC: Healthy control, NC: Negative control, Diclo: Diclofenac sodium, Dasha: *Dashamoola Kwatha*, DF: *Dashamoola* Formulation. Values are expressed as Mean ± SEM (*n* = 3). Comparisons were done between NC/HC and each individual treated group by Dunnett’s multiple comparison test

NC group rats showed up-regulation (~56.3-fold) in the abundance of iNOS mRNA, compared to levels in HC. Treatment with Diclo and Dasha resulted in down-regulation (~1.1-fold and ~2.3-fold respectively) of iNOS expression as compared to NC. DF1911, DF2112 and DF2813 showed down-regulation of iNOS gene expression by ~1.2-fold, ~1.8-fold and ~1.1-fold respectively when compared to NC (Fig. 7).

## Discussion

The alarming safety and efficacy issues of many drugs used for the treatment of inflammatory disorders have continued since years. There always has been focus on the biologically active compounds from plants and efficacy of natural products against inflammatory disorders [9, 21, 22].

In the present study we investigated the herbal preparations with code names DF1911, DF2112 and DF2813; and compared their anti-inflammatory effect with Diclofenac sodium and routinely practiced *Dashamoola Kwatha* formulation, which is commonly used for the treatment of inflammatory disorders. The CFA induced experimental model has been extensively used in the study of inflammatory processes and for evaluation of anti-inflammatory agents [23, 24]. CFA administration into hind paw of rats leads to marked swelling which persists for weeks as a primary reaction [25]. After a few days, the delayed systemic response is also seen in the form of controlateral paw as well as front paw swelling [26, 27].

In the present study, the increase in paw volume seen in the NC group is believed to be produced due to elevated immune response during the late phase of the model [28]. There was a decrease in paw volume seen in Diclo, Dasha as well as test formulation treated groups, except for DF2813 at a dose of 500 and 1000 mg/kg bw. However, in the DF2813 treated groups at a dose of 500 and 1000 mg/kg bw, there was a slight increase in paw volume from day 21 to 28. In comparison with NC group, the significant (*p* < 0.05) decrease in paw volume of rats in DF1911 and DF2112 treated groups at both doses of 250 and 500 mg/kg bw suggests intervention of these formulations in the inflammatory pathways. These results are comparable to Diclofenac sodium (15 mg/kg bw) treated animals, which had 60.53% inhibition of paw edema.

Changes in body weight have also been assessed to study the response to the therapy of anti-inflammatory drugs. The increase in body weight during the treatment of standard drug and test formulations may be due to restorations of the absorption capacity of the intestine which has been reduced to be reduced in NC animals [29].

From the results, it is clear that decrease in haemoglobin level and RBC count represents anemic condition in NC group rats. Dicofenac sodium, *Dashamoola Kwatha* and test formulation treated groups restored these levels back to normal. Increase in WBC count plays an important role in body defence mechanism. It may be due to release of interleukins, responsible for production of both granulocytes and macrophages colony stimulating factor [30]. In the present study, migration of leukocytes into inflamed area was suppressed by standard drug and test formulations as seen from decrease in total WBC count, which indicates recovery from the inflammatory condition [31]. ESR is an estimate of the suspension stability of RBCs in plasma, related to the number and size of red cells and to the relative concentration of plasma proteins especially fibrinogen and α and β globulins. Increase in ESR is an indication of active but obscure disease processes [32]. Increased ESR is a common diagnostic feature in inflammatory conditions. The ESR level which was markedly elevated in NC group rats was decreased by test formulations and the effect was comparable to the standard drug.

It is postulated that during the process of inflammation, the release of inflammatory mediators such as histamine, bradykinin and prostaglandins causes increase in vascular permeability of tissues to albumin which leads to reduction of albumin levels in serum [33]. In the present study, there was significant (*p* < 0.001) decrease in albumin levels in NC group rats as compared to HC. Treatment with DF2112 and DF2813 formulations significantly increased albumin levels in rats which indicated that DF2112 and DF2813 might have a suppressive action on inflammatory mediators.

Lysosomal enzymes play an important role in the development of acute and chronic inflammation [31]. These enzymes are the indicators of the phagocytic activity that can be used as sensitive markers of cellular integrity and toxicity induced by different pathological conditions. Therefore, inhibition of release of lysosomal enzymes or stabilization of lysosomal membranes is important for the anti-inflammatory process of the drug [31]. In the present study, DF1911 and DF2112 considerably decreased the levels of lysosomal enzyme activity viz. transaminases (SGOT, SGPT) and alkaline phosphatase. Acid phosphatase is also an important marker for the examination of the integrity of lysosomal membrane, as it is responsible for tissue damage. The increased levels of acid phosphatise in NC group rats can be attributed to persistent inflammation. Significant decrease in the levels of acid phosphatase in DF1911-MD, DF2112-LD and DF2112-HD treated groups shows their anti-inflammatory effects in CFA induced inflammation in rats.

Lipid peroxidation was determined by measuring TBARS produced during lipid peroxidation of polyunsaturated fatty acids in cell membranes. An increased level of MDA is a marker of lipid peroxidation due to oxidative damage. MDA levels in NC group treated animals could be linked to generation of free radicals, resulting in membrane lipid peroxidation. MDA is an end product of lipid peroxidation which is used as an indicator of tissue damage brought about by series of chain reactions [34, 35]. In the present study, NC group animals showed highest MDA level (61.7 ± 3.69 μM/g of tissue) which is evident of the high level of lipid peroxidation caused by CFA. However, significant decrease in MDA levels was observed in rats treated with Diclofenac sodium, DF1911-HD, DF2112-LD, DF2112-MD, DF2112-HD, DF2813-MD and DF2813-HD which shows the most promising recovery effect from liver tissue damage.

The inhibition of the increase in hind paw volume may be associated with inhibition of neutrophil infiltration [36], which is also supported by histological studies of paw tissues. Severe neutrophil infiltration is seen in paw tissues of NC group rats and on treatment with DF1911, DF2112 and DF2318 there is reduction in the neutrophil infiltration but DF1911 and DF2112 formulations at a doses of 250 and 500 mg/kg bw showed a maximum reduction in neutrophil infiltration. Both these formulations have suppressed the macrophage infiltration and edema in paw tissues. Macrophages are the phagocytic cells known to play key roles in the inflammatory process. These cells have destructive effects during the onset of inflammation when they are activated.

Along with paw volume measurement and biochemical parameters, the expression of key cytokines viz. IL1-β, IL-6 and, TNF-α iNOS was also studied as these cytokines have been reported to be expressed at significant levels in the chronic state of the inflammatory diseases [20]. We found that the test formulations decreased the concentration of the pro-inflammatory cytokines IL1-β, IL-6 and TNF-α at the local inflammation site in the animal model. These cytokines are crucially important in rats as well as in humans as they contribute to many features of tissue inflammation or inflammatory conditions [37, 38]. The Nos2, which encodes the enzyme nitric oxide synthase 2 (inducible nitric oxide synthase) is also concerned with the inflammatory responses [39]. The large amount of NO produced plays a key role in the pathogenesis of inflammatory conditions [40]. Nos2 was found to be down-regulated in the test formulation treated groups.

To conclude, DF1911 and DF2112 at a dose of 500 mg/kg bw showed a marked reduction in paw edema volume, improved blood indices, normalized the hematological and biochemical abnormalities. Further, histopathological studies confirmed anti-inflammatory effect of DF1911 and DF2112 formulations in CFA induced inflammation. The treatments revealed improvements in paw tissue histology. These treatments also lowered expression of inflammatory biomarkers. DF1911 and DF2112 (500 mg/kg bw) were substantially more effective than *Dashamoola Kwatha* formulation. In summary, results of the present study indicate beneficial therapeutic effects of DF1911 and DF2112, which reduced inflammation in CFA induced inflammation model. Anti-inflammatory activity of individual plants could be enhanced in formulation due to their synergistic effect. In summary, the DF2112 formulation possessed highest anti-inflammatory activity evident in CFA induced inflammation in rat model.

## Conclusions

In conclusion, we evaluated the efficacy of orally administered DF1911, DF2112 and DF2813, containing the mixtures of plants, in Complete Freund’s Adjuvant induced inflammation model in rats. This study provides strong evidence for promising anti-inflammatory activity of DF1911 and DF2112. Thus, DF1911 and DF2112 could potentially be considered as an alternative therapy from natural sources for treatment of inflammation. However, further preclinical and clinical studies are necessary.

## Abbreviations

ALP: Alkaline phosphatase
AM: *A. marmelos*
AR: *A. racemosus*
CFA: Complete Freund’s adjuvant
CL: *C. longa*
Dasha: *Dashamoola Kwatha*
DF: *Dashamoola* formulation
DG: *D. gangeticum*
Diclo: Diclofenac sodium
GA: *G. arborea*
H&E: Hematoxylin and Eosin
HC: Healthy control
HD: Higher dose
LD: Lower dose
MD: Middle dose
MDA: Malondialdehyde
NC: Negative control
OI: *O. indicum*
PO: *P. obtusifolia*
PP: *P. pinnata*
SA: *S. anguivi*
SC: *S. colais*
SGOT: Serum glutamic-oxaloacetic transaminase
SGPT: Serum glutamic-pyruvic transaminase
SV: *S. virginianum*
TBARS: Thiobarbituricacid-reactive substances
TC: *T. chebula*
TT: *T. terrestris*
UP: *U. picta*
